# Risk Factors for Acquiring Scrub Typhus among Children in Deoria and Gorakhpur Districts, Uttar Pradesh, India, 2017

**DOI:** 10.3201/eid2412.180695

**Published:** 2018-12

**Authors:** Jeromie Wesley Vivian Thangaraj, Ravi Vasanthapuram, Leonard Machado, Govindakarnavar Arunkumar, Samir V. Sodha, Kamran Zaman, Tarun Bhatnagar, Shafeeq K. Shahul Hameed, Arun Kumar, Jazeel Abdulmajeed, Anoop Velayudhan, Avinash Deoshatwar, Anita S. Desai, K. Hemanth Kumar, Nivedita Gupta, Kayla Laserson, Manoj Murhekar, Scrub Typhus Risk Factor Study Group

**Affiliations:** Indian Council of Medical Research National Institute of Epidemiology, Chennai, India (J.W.V. Thangaraj, T. Bhatnagar, M. Murhekar);; National Institute of Mental Health and Neurosciences, Bengaluru, India (R. Vasanthapuram, S.K.S. Hameed, A.S. Desai);; World Health Organization, New Delhi, India (L. Machado, A. Kumar);; Manipal Centre for Virus Research, Manipal Academy of Higher Education (Deemed to be University), Manipal, India (G. Arunkumar, J. Abdulmajeed, K.H. Kumar);; US Centers for Disease Control and Prevention, New Delhi (S.V. Sodha, A. Velayudhan, K. Laserson);; Indian Council of Medical Research National Institute of Virology, Gorakhpur, India (K. Zaman);; Indian Council of Medical Research National Institute of Virology, Pune, India (A. Deoshatwar);; Indian Council of Medical Research, New Delhi (N. Gupta)

**Keywords:** acute encephalitis syndrome, scrub typhus, risk factors, Deoria, Gorakhpur, Uttar Pradesh, India, rickettsial diseases, bacteria, bacterial infection, Orientia tsutsugamushi, zoonoses

## Abstract

Scrub typhus is associated with outbreaks of acute encephalitis syndrome in Uttar Pradesh, India. A case-control study indicated that children residing, playing, or visiting fields; living with firewood stored indoors; handling cattle fodder; and practicing open defecation were at increased risk for scrub typhus. Communication messages should focus on changing these behaviors.

Outbreaks of acute encephalitis syndrome (AES) with high case-fatality rates have been reported from Gorakhpur district, Uttar Pradesh, India, for >2 decades. These outbreaks occur during monsoon and postmonsoon seasons and predominantly affect children. Scrub typhus (ST) accounted for about two thirds of AES cases (*1**–**4*) and is also an important etiology of acute febrile illness (AFI) in the region (*5*). Untreated cases of ST-attributable AFI can progress to AES.

ST, caused by the bacterium *Orientia tsutsugamushi*, is transmitted by the bite of trombiculid mites, which live in moist soil covered with vegetation (*6*). Several risk factors, including certain household characteristics, work-related practices, and behaviors, have been identified among adult ST patients (*7*–*11*). Household characteristics include location of the house near a grassland, vegetable field, or ditch; presence of mud floors; piled weeds inside the house; and scrub vegetation in the vicinity (*7*). Work-related practices include working in vegetable fields or hilly areas and working in short sleeves or with bare hands (*8*). Certain behaviors such as lying on grass and squatting to defecate or urinate also are associated with ST (*8*,*10*,*11*). Because these risk factors are region specific, we conducted an exploratory case-control study among children in Gorakhpur and Deoria districts of Uttar Pradesh to identify factors associated with ST infection.

## The Study

We conducted AFI surveillance in public health facilities in Deoria (n = 5) and Gorakhpur (n = 3) districts during October 3–November 11, 2017, a period coinciding with AES outbreaks in Gorakhpur district. We enrolled children 2–15 years of age with a >3-day history of fever, from whom we collected 2 mL of blood after obtaining written informed consent from parents and assent from children 7–15 years of age. We screened serum samples for IgM and IgG against *O. tsutsugamushi* by using ELISA kits (Scrub Typhus Detect; InBios International Inc., Seattle, WA, USA). For our study, an optical density value >0.5 indicated IgM positivity. This cutoff has 93% sensitivity and 91% specificity for ST diagnosis (*12*). An optical density value <0.5 indicated IgM and IgG negativity.

Febrile children who were positive for *O. tsutsugamushi* IgM were considered case-patients, whereas patients who were seronegative for IgM and IgG were considered controls. Case-patients and controls and their parents or guardians were interviewed in their houses by using a pretested structured questionnaire to collect information on sociodemographics, household characteristics, behaviors, and environmental exposures during the 2 weeks before fever onset. Interviewers were blinded to the case-patient or control status of children except during the first week of study. 

We calculated crude odds ratios (ORs) and 95% CIs associated with different exposures. We included variables with p value <0.2 in univariate analysis in a stepwise backward elimination model to identify variables for inclusion in the final unconditional multiple logistic regression model by using Stata 13 (StataCorp LLC, College Station, TX, USA).

We recruited 819 AFI patients, of whom 155 (18.9%) had *O. tsutsugamushi* IgM (case-patients) and 409 (49.9%) were seronegative (controls) (Technical Appendix Table 1). We excluded 255 (31.1%) patients who had only *O. tsutsugamushi* IgG. We interviewed all case-patients and 406 controls. All case-patients and controls were from rural areas. The mean age of case-patients was higher than controls (7.0 vs. 6.3 years; p = 0.018); 39% of case-patients and 51% of controls were <5 years of age. A higher proportion of case-patients than controls were from households that owned agricultural land (83% vs. 73%; p = 0.017) (Table 1). 

**Table 1:** Sociodemographic details of cases and controls, Deoria and Gorakhpur districts, Uttar Pradesh, 2017

Variables	% Case-patients, n = 155	% Controls, n = 406	p value
Age group, y			
2–5	39	51	
6–10	46	35	
11–15	14	15	
Mean age, y (SD)	7.0 (3.2)	6.3 (3.5)	0.0176
Sex			
M	61	61	
F	39	39	0.882
District			
Deoria	56	55	
Gorakhpur	44	45	
Religion			
Hindu	92	89	
Other	8	11	0.348
Median duration of fever, d (IQR)	7 (4.5–10.0)	5 (4–8)	
Household owns agricultural land			
No	17	27	
Yes	83	73	0.017

*Values are percentages unless otherwise indicated. IQR, interquartile range.

Aside from fever, clinical signs and symptoms among patients varied (Technical Appendix Table 2). Five ST patients, who did not receive doxycycline/azithromycin, had onset of neurologic manifestations and required hospitalization; they recovered after administration of azithromycin.

Univariate analysis showed that ST patients were more likely than controls to live in houses located within or adjoining fields, houses with unpaved surroundings with mud or unkempt grass, and mud-floored houses (Table 2). In addition, a higher proportion of case-patients lived in households that used wood or cow dung for cooking and stored this fuel indoors. Compared with controls, children with ST were more likely to have played in agricultural fields, defecated in agricultural fields, bathed in a river or stream, carried grass bundles on their heads, handled fodder for cattle, or visited or accompanied parents to an agricultural field during the 2 weeks before fever onset (Table 2).

**Table 2:** Risk factors associated with scrub typhus, Gorakhpur and Deoria districts, Uttar Pradesh, 2017*

Risk factor	% Cases, n = 155	% Controls, n = 406	OR (95% CI)	p value	aOR (95% CI)
Location of house within or adjoining field	67	52	1.86 (1.26–2.74)	0.002	1.56 (1.02–2.38)
Mud or grassy approach road to house	41	36	1.21 (0.82–1.76)	0.335	
Mud or grassy pavement in front of house	81	71	1.73 (1.09–2.74)	0.018	
Mud house floor	72	59	1.88 (1.25–2.82)	0.002	
Presence of waterbody within 100 m of house	61	51	1.52 (1.04–2.23)	0.029	
Location of toilet					
No toilet	76	57	2.76 (1.59–4.78)	0.001	
Within house	12	24	1		
Outside house	12	18	1.36 (0.66–2.80)		
Wood or dung fuel	55	34	2.32 (1.59–3.39)	<0.01	
Storage of fuel (wood or dung) inside house or veranda	53	34	2.16 (1.48–3.14)	<0.01	1.61 (1.06–2.45)
Food waste given to cattle or disposed in common place	30	25	1.27 (0.84–1.92)	0.248	
Spotted rats inside house, daily	80	79	1.09 (0.68–1.73)	0.731	
Spotted rats outside house, daily	43	48	0.81 (0.56–1.17)	0.263	
Presence of scrub vegetation around house	79	73	1.38 (0.88–2.17)	0.154	
Storage of wet food produce inside house or veranda	37	25	1.78 (1.20–2.65)	0.004	
Storage of dried food produce inside house or veranda	83	72	1.87 (1.17–2.99)	0.009	
Livestock kept inside house or veranda	32	22	1.65 (1.09–2.48)	0.017	
Fodder for livestock stored inside house or veranda	32	24	1.49 (0.99–2.24)	0.055	
Place of drying clothes; bushes, ground, fence, or roof	30	25	1.30 (0.86–1.95)	0.213	
Usual clothing at home for lower body; fully covered	24	21	1.18 (0.76–1.83)	0.451	
Usual clothing at home for upper body; fully covered	8	11	0.71 (0.36–1.38)	0.308	
Usual clothing for school for lower body; fully covered	48	42	1.14 (0.74–1.76)	0.543	
Usual clothing for school for upper body; fully covered	51	43	1.27 (0.82–1.97)	0.283	
Usual clothing during playing for lower body; fully covered	21	17	1.26 (0.79–1.99)	0.334	
Usual clothing during playing for upper body; fully covered	8	8	1.0 (0.51–1.97)	0.980	
Using clothing while sleeping for lower body; fully covered	19	17	1.1 (0.68–1.78)	0.692	
Using clothing while sleeping for upper body; fully covered	7	7	0.89 (0.42–1.88)	0.763	
Change of clothes before sleep; never or sometimes	86	84	1.24 (0.73–2.11)	0.423	
Do not wear footwear during playing	73	65	1.25 (0.82–1.89)	0.298	
Do not wear footwear while going to school	6	4	1.37 (0.59–3.19)	0.465	
Place of play in 2 wks before illness					
Indoor	5	17	1	0.001	
Around house	77	76	3.77 (1.68–8.45)		2.68 (1.15–6.27)
Agricultural fields	18	7	9.38 (3.68–23.90)		5.20 (1.92–14.15)
Practice of washing after playing					
Usually takes bath	13	14	1		
Usually washes hands or feet	59	60	1.05 (0.60–1.84)	0.769	
None	28	25	1.21 (0.65–2.25)	0.225	
Floor as usual place of sleeping	14	11	1.41 (0.81–2.44)		
Place of defecation in 2 wks before illness					
Around house	7	14	1.12 (0.52–2.42)	0.001	0.93 (0.41–2.12)
Field	76	48	3.62 (2.25–5.82)		2.00 (1.18–3.39)
Toilet	17	38	1		1
Early morning defecation	30	25	1.28 (0.85–1.93)	0.237	
Swam or bathed in river or stream in 2 wks before illness	11	5	2.37 (1.21–4.67)	0.012	1.74 (0.80–3.77)
Visited field in 2 wks before illness	61	39	2.49 (1.70–3.63)	<0.01	1.65 (1.07–2.52)
Carried bundle of grass over head in 2 wks before illness	14	6	2.29 (1.25–4.21)	<0.01	0.75 (0.35–1.61)
Handled cattle fodder in 2 wks before illness	25	9	3.46 (2.1–5.69)	<0.01	2.05 (1.13–3.70)

*aOR, adjusted odds ratio; OR, odds ratio.

Multivariable analysis showed that children residing in houses within or adjoining fields (adjusted OR [aOR] 1.56, 95% CI 1.02–2.38) and that stored firewood indoors (aOR 1.61, 95% CI 1.06–2.45) had higher odds of acquiring ST. Open defecation (aOR 2.0, 95% CI 1.18–3.39), playing in (aOR 5.2, 95% CI 1.92–14.2) or visiting (aOR 1.65, 95% CI 1.07–2.52) agricultural fields, and handling cattle fodder (aOR 2.05, 95% CI 1.13–3.70) also were associated with ST (Table 2).

## Conclusions

In Gorakhpur and Deoria districts, recent exposure to the outdoor environment, either while defecating or playing in agricultural fields, as well as visiting agricultural fields, storing firewood indoors, and handling fodder for cattle were associated with higher risk for acquiring ST among children. Of these risk factors, defecation in the agricultural field was the most common exposure at the population level. The observed association of ST and defecation in fields is consistent with the findings from an ST outbreak in Manipur, India, where persons who defecated or urinated in the jungle or bushy areas were found to be at higher risk for ST (*11*). In Gorakhpur and Deoria districts, about one third of the study population had toilets, a finding consistent with the 2015 national level survey, which indicated that 29.5% of the households in rural Uttar Pradesh had sanitary toilets and 27.3% of households that had access to toilets were using them (http://www.mdws.gov.in/sites/default/files/Swachh%20Survekshan%20Report%20Eng.PDF). Efforts to prevent ST in the region therefore also need to focus on constructing household and community toilets, as well as behavior change communication about avoiding open defecation in the fields. In addition, storing firewood and fodder indoors attracts rodents that can harbor mites. Mites present on the firewood and fodder collected from fields might expose children to mite bites during storage or handling of the cattle fodder.

Our study had limitations. First, although OT IgM is detectable as early as 4 days after fever onset (*13*), some ST patients in our study might have been negative for IgM and hence misclassified as controls. However, such nondifferential misclassification is likely to underestimate the actual association. Further, the median duration of fever was 7 days (interquartile range 4.5–10.0 days) among case-patients and 5 days (interquartile range 4–8 days) among controls (Table 1), indicating minimal possibility of such misclassification. Some controls might have been misclassified as case-patients because of persistence of IgM from previous infection (*14*). However, this possibility is less likely considering that IgM peaks by 4 weeks of infection and declines rapidly thereafter (*15*). Second, our control-patients were AFI patients from healthcare facilities and not healthy children from the general population. Nevertheless, the behaviors of children visiting healthcare facilities before their febrile illness and children from the general population are less likely to be different.

For prevention of ST among children in Gorakhpur and Deoria districts, communication messages should focus on changing behaviors such as defecating or playing in agricultural fields and unnecessary visits to agricultural fields. Onset of central nervous system manifestations among untreated ST patients underscores the importance of early administration of doxycycline/azithromycin to ST patients in Gorakhpur and Deoria districts to prevent progression to AES.

Technical Appendix Additional details on risk factors for acquisition of scrub typhus among children, Uttar Pradesh, India, 2017.

## References

[1] Murhekar MV, Mittal M, Prakash JAJ, Pillai VM, Mittal M, Girish Kumar CP, et al. Acute encephalitis syndrome in Gorakhpur, Uttar Pradesh, India - Role of scrub typhus. J Infect. 2016;73:623–6. 10.1016/j.jinf.2016.08.014 27592263

[2] Mittal M, Thangaraj JWV, Rose W, Verghese VP, Kumar CPG, Mittal M, et al. Scrub typhus as a cause of acute encephalitis syndrome, Gorakhpur, Uttar Pradesh, India. Emerg Infect Dis. 2017;23:1414–6. 10.3201/eid2308.170025 28726617 PMC5547812

[3] Pulla P. Disease sleuths unmask deadly encephalitis culprit. Science. 2017;357:344. 10.1126/science.357.6349.344 28751590

[4] Mittal M, Bondre V, Murhekar M, Deval H, Rose W, Verghese VP, et al. Acute encephalitis syndrome in Gorakhpur, Uttar Pradesh, 2016: clinical and laboratory findings. Pediatr Infect Dis J. 2018;37:1101–6. 10.1097/INF.0000000000002099 29746378

[5] Vivian Thangaraj JW, Mittal M, Verghese VP, Kumar CPG, Rose W, Sabarinathan R, et al. Scrub typhus as an etiology of acute febrile illness in Gorakhpur, Uttar Pradesh, India, 2016. Am J Trop Med Hyg. 2017;97:1313–5. 10.4269/ajtmh.17-0135 28820712 PMC5817754

[6] Rahi M, Gupte MD, Bhargava A, Varghese GM, Arora R. DHR-ICMR Guidelines for diagnosis & management of Rickettsial diseases in India. Indian J Med Res. 2015;141:417–22. 10.4103/0971-5916.159279 26112842 PMC4510721

[7] Lyu Y, Tian L, Zhang L, Dou X, Wang X, Li W, et al. A case-control study of risk factors associated with scrub typhus infection in Beijing, China. PLoS One. 2013;8:e63668–63668. 10.1371/journal.pone.0063668 23691083 PMC3653850

[8] Kweon S-S, Choi J-S, Lim H-S, Kim J-R, Kim K-Y, Ryu S-Y, et al. A community-based case-control study of behavioral factors associated with scrub typhus during the autumn epidemic season in South Korea. Am J Trop Med Hyg. 2009;80:442–6. 10.4269/ajtmh.2009.80.442 19270296

[9] Trowbridge P, P D, Premkumar PS, Varghese GM. Prevalence and risk factors for scrub typhus in South India. Trop Med Int Health. 2017;22:576–82. 10.1111/tmi.12853 28173608

[10] Varghese GM, Raj D, Francis MR, Sarkar R, Trowbridge P, Muliyil J. Epidemiology & risk factors of scrub typhus in south India. Indian J Med Res. 2016;144:76–81. 10.4103/0971-5916.193292 27834329 PMC5116902

[11] Singh SI, Devi KP, Tilotama R, Ningombam S, Gopalkrishna Y, Singh TB, et al. An outbreak of scrub typhus in Bishnupur district of Manipur, India, 2007. Trop Doct. 2010;40:169–70. 10.1258/td.2010.090468 20555047

[12] Blacksell SD, Tanganuchitcharnchai A, Nawtaisong P, Kantipong P, Laongnualpanich A, Day NPJ, et al. Diagnostic accuracy of the InBios Scrub Typhus Detect enzyme-linked immunoassay for the detection of IgM antibodies in Northern Thailand. Clin Vaccine Immunol. 2015;23:148–54. 10.1128/CVI.00553-15 26656118 PMC4744921

[13] Ching WM, Wang H, Eamsila C, Kelly DJ, Dasch GA. Expression and refolding of truncated recombinant major outer membrane protein antigen (r56) of *Orientia tsutsugamushi* and its use in enzyme-linked immunosorbent assays. Clin Diagn Lab Immunol. 1998;5:519–26. 9665960 10.1128/cdli.5.4.519-526.1998 PMC95611

[14] Varghese GM, Rajagopal VM, Trowbridge P, Purushothaman D, Martin SJ. Kinetics of IgM and IgG antibodies after scrub typhus infection and the clinical implications. Int J Infect Dis. 2018;71:53–5. 10.1016/j.ijid.2018.03.018 29653201 PMC5985369

[15] Kim DM, Lee Y-M, Back J-H, Yang TY, Lee JH, Song H-J, et al. A serosurvey of *Orientia tsutsugamushi* from patients with scrub typhus. Clin Microbiol Infect. 2010;16:447–51. 10.1111/j.1469-0691.2009.02865.x 19778303

