# Supplementary material for: Risk Factors for Acquiring Scrub Typhus among Children in Deoria and Gorakhpur Districts, Uttar Pradesh, India, 2017
Source: Emerg Infect Dis. 2018 Dec;24(12):2364–7. doi: 10.3201/eid2412.180695 (PMC6256400; doi:10.3201/eid2412.180695)
Supplement: Technical Appendix — Additional details on risk factors for acquisition of scrub typhus among children, Uttar Pradesh, India, 2017. [file 18-0695-Techapp-s1.pdf]

Article DOI: https://doi.org/10.3201/eid2412.180695

Risk Factors for Acquisition of Scrub Typhus among Children in Deoria and Gorakhpur Districts, Uttar Pradesh, India, 2017

Technical Appendix

Technical Appendix Table 1. Details of AFI patients enrolled, tested for IgM and IgG antibodies against *O. tustsugamushi* (OT), and interviewed.

Characteristic	Gorakhpur	Deoria	Total
AFI patients enumerated	372	447	819
Positive for IgM antibodies against OT (cases), no. (%)	68 (18.3)	87 (19.5)	155
Negative for IgM and IgG against OT (controls), no. (%)	187 (50.3)	222 (49.7)	409
Positive for IgG antibodies against OT alone	117	138	255
Total cases and controls	255	309	564
Cases interviewed	68	87	155
Controls interviewed	184	222	406
Cases and controls interviewed	252	309	561

Technical Appendix Table 2. Clinical features of cases and controls, Gorakhpur and Deoria districts, 2017

Symptom or sign	% Cases, n = 155	% Controls, n = 406	p value
Headache	54	59	0.16
vomiting	34	26	0.05
Loose motion	15	12	0.19
Nasal discharge	20	36	<0.001
Skin rash	8	10	0.22
Abdominal pain	27	22	0.11
cough	45	58	<0.001
Pain in limbs	20	24	0.14
Dehydration	14	9	0.04
Periorbital odema	8	7	0.44
Conjunctival congestion	10	10	0.44
Hepatomegaly	6	3	0.04
Icterus	15	17	0.23
Odema	1	3	0.16
Lymphadenopathy	15	10	0.03
Splenomegaly	4	2	0.18
Skin rash	3	4	0.35
Eschar	2	1	0.11
